# Sepsis-Associated Acute Kidney Injury: Where Are We Now?

**DOI:** 10.3390/medicina60030434

**Published:** 2024-03-06

**Authors:** Dimitris Kounatidis, Natalia G. Vallianou, Sotiria Psallida, Fotis Panagopoulos, Evangelia Margellou, Dimitrios Tsilingiris, Irene Karampela, Theodora Stratigou, Maria Dalamaga

**Affiliations:** 1 Department of Internal Medicine, Evangelismos General Hospital, 45-47 Ipsilantou Str., 10676 Athens, Greece; dimitriskounatidis82@outlook.com (D.K.); fotis_1992@hotmail.com (F.P.); evmargellou@gmail.com (E.M.); theodorastratigou@yahoo.gr (T.S.); 2 Department of Microbiology, “KAT” General Hospital of Attica, 14561 Athens, Greece; sotiriapsallida@gmail.com; 3 Department of Internal Medicine, Democritus University of Thrace, Dragana, 68100 Alexandroupoli, Greece; tsilingirisd@gmail.com; 4 Second Department of Critical Care, Attikon University Hospital, 1 Rimini Str., 12462 Athens, Greece; eikaras1@gmail.com; 5 Department of Biological Chemistry, National and Kapodistrian University of Athens, 75 Mikras Asias Str., 11527 Athens, Greece

**Keywords:** acute kidney injury, biomarkers, infection, septic shock, sepsis-associated acute kidney injury, sepsis, therapy

## Abstract

Worldwide, sepsis is a well-recognized cause of death. Acute kidney injury (AKI) may be related to sepsis in up to 70% of AKI cases. Sepsis-associated AKI (SA-AKI) is defined as the presence of AKI according to the Kidney Disease: Improving Global Outcomes criteria in the context of sepsis. SA-AKI is categorized into early, which presents during the first 48 h of sepsis, and late, presenting between 48 h and 7 days of sepsis. SA-AKI is associated with a worse prognosis among patients with sepsis. However, there are different SA-AKI phenotypes as well as different pathophysiological pathways of SA-AKI. The aim of this review is to provide an updated synopsis of the pathogenetic mechanisms underlying the development of SA-AKI as well as to analyze its different phenotypes and prognosis. In addition, potential novel diagnostic and prognostic biomarkers as well as therapeutic approaches are discussed. A plethora of mechanisms are implicated in the pathogenesis of SA-AKI, including inflammation and metabolic reprogramming during sepsis; various types of cell death such as apoptosis, necroptosis, pyroptosis and ferroptosis; autophagy and efferocytosis; and hemodynamic changes (macrovascular and microvascular dysfunction). Apart from urine output and serum creatinine levels, which have been incorporated in the definition of AKI, several serum and urinary diagnostic and prognostic biomarkers have also been developed, comprising, among others, interleukins 6, 8 and 18, osteoprotegerin, galectin-3, presepsin, cystatin C, NGAL, proenkephalin A, CCL-14, TIMP-2 and L-FABP as well as biomarkers stemming from multi-omics technologies and machine learning algorithms. Interestingly, the presence of long non-coding RNAs (lncRNAs) as well as microRNAs (miRNAs), such as PlncRNA-1, miR-22-3p, miR-526b, LncRNA NKILA, miR-140-5p and miR-214, which are implicated in the pathogenesis of SA-AKI, may also serve as potential therapeutic targets. The combination of omics technologies represents an innovative holistic approach toward providing a more integrated view of the molecular and physiological events underlying SA-AKI as well as for deciphering unique and specific phenotypes. Although more evidence is still necessary, it is expected that the incorporation of integrative omics may be useful not only for the early diagnosis and risk prognosis of SA-AKI, but also for the development of potential therapeutic targets that could revolutionize the management of SA-AKI in a personalized manner.

## 1. Introduction

Sepsis represents a serious condition and a significant cause of death in hospitals. According to the Third International Consensus Definitions for Sepsis and Septic Shock, sepsis is defined as a “life-threatening organ dysfunction caused by a dysregulated host response to infection” [1]. Septic shock is defined as sepsis that requires vasopressors to maintain a mean arterial pressure (MAP) of at least 65 mmHg together with serum lactate levels of at least 2 mmol/L (18 mg/dL) in the absence of hypovolemia [1]. Septic shock has been related to a mortality rate of more than 40% [1,2]. As sepsis incidence is on the rise, there is an increasing interest in sepsis and its complications, such as sepsis-associated acute kidney injury (SA-AKI) [3].

AKI has been defined as an increase in serum creatinine levels of at least 50% within 7 days or an increase in serum creatinine of at least 0.3 mg/dL within 2 days or a decrease in the urine output (oliguria) within at least 6 h. This AKI definition is according to the 2020 Consensus of KDIGO (Kidney Disease: Improving Global Outcomes) [4]. SA-AKI is AKI as defined by the KDIGO criteria, which is documented to occur within 7 days of the diagnosis of sepsis, as defined by the above-mentioned SEPSIS-3 criteria [1,2,3,4]. The current definition of SA-AKI has just been suggested by the ADQI (Acute Disease and Quality Initiative) Group in 2023 [5]. SA-AKI is further classified as early SA-AKI, documented within 48 h, and late SA-AKI, documented between 48 h and 7 days of sepsis onset [5]. SA-AKI has been associated with worse prognoses when compared with the presence of sole sepsis or AKI [6,7]. However, due to the lack of consensus definitions regarding SA-AKI until very recently, estimations cannot be too accurate.

When considering the diagnosis of SA-AKI, it is essential to differentiate it from other potential causes of AKI, particularly those unrelated to sepsis. Some of the key differential diagnoses of SA-AKI include (1) pre-renal azotemia that can result from conditions such as dehydration, heart failure, or severe blood loss; (2) acute tubular necrosis due to ischemia or exposure to nephrotoxic substances such as certain medications (e.g., NSAIDs, contrast agents, aminoglycoside antibiotics); (3) interstitial nephritis often caused by medications (e.g., antibiotics, proton pump inhibitors, NSAIDs) or autoimmune diseases; (4) glomerulonephritis; (5) obstructive uropathy due to conditions such as kidney stones, tumors, or benign prostatic hyperplasia; (6) hepatorenal syndrome that occurs as a complication of severe liver dysfunction; (7) thrombotic microangiopathy in conditions such as thrombotic thrombocytopenic purpura or hemolytic uremic syndrome; (8) acute interstitial nephritis, often triggered by medications, infections, or autoimmune diseases; (9) toxic nephropathy due to exposure to various toxins or heavy metals causing direct damage to the kidneys; (10) rhabdomyolysis; and (11) vasculitis [8]. The diagnosis of SA-AKI involves careful clinical evaluation, laboratory tests (including, among others, serum creatinine and urine output measurements, blood cultures, etc.), and the consideration of the patient’s medical history and clinical context. Often, distinguishing the underlying cause of AKI requires a combination of clinical judgment, laboratory investigations, imaging studies, and sometimes kidney biopsy [5,9]. The timely diagnosis of SA-AKI is of utmost importance. Interventions, such as a balanced fluid administration and the initiation of vasopressors to maintain an adequate MAP together with the avoidance of nephrotoxic substances may result in the resolution of SA-AKI. In addition, the initiation of kidney replacement therapy (KRT) should not be overlooked [10]. Last but not least, the prompt administration of antibiotics may be lifesaving in the setting of sepsis. Lately, there is a growing interest and ongoing research in the field of SA-AKI with the emergence of potential novel therapeutic targets based upon gene expression profiling and bioinformatics. 

The aim of this review is to provide an updated synopsis of pathogenetic mechanisms underlying the development of SA-AKI as well as to analyze its different phenotypes and prognosis. In addition, potential novel diagnostic and prognostic biomarkers as well as therapeutic approaches are discussed based on the pathophysiology of SA-AKI and current available data.

## 2. Methodology of Literature Search

**Figure 1:**
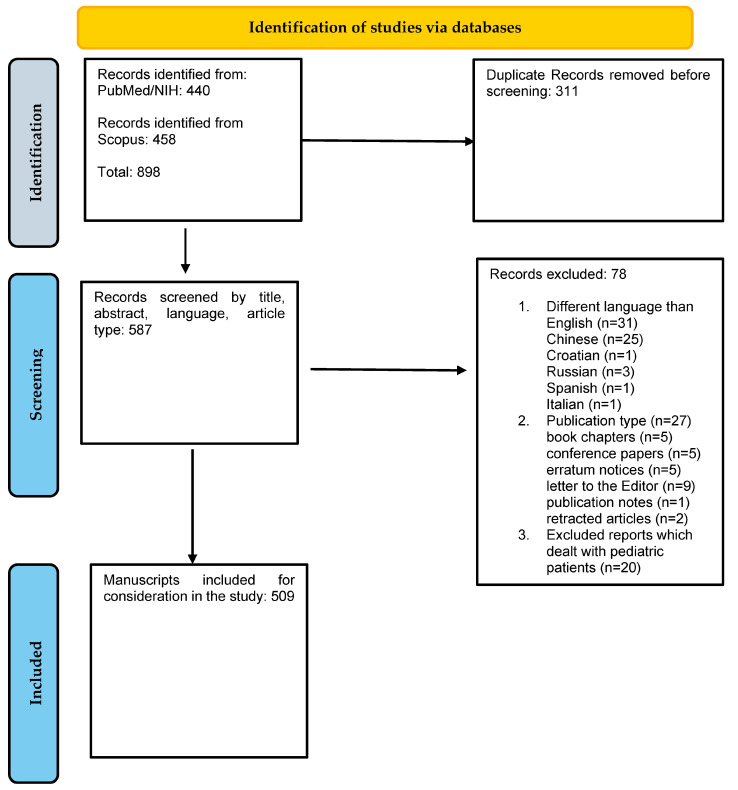
Flowchart depicting the steps of the literature search.

Although this manuscript is not a systematic review, for its preparation, we applied the same general term “sepsis-associated acute kidney injury” in two major biomedical databases (PubMed NIH and Scopus) from 1 January 2014 to 31 December 2023. DT, IK and MD performed the literature search, which yielded 898 related research items indexed in these databases. After the exclusion of 311 duplicate items, we screened 587 records. Among these, we excluded 31 publications that were written in Chinese (*n* = 25), Croatian (*n* = 1), Russian (*n* = 3), Spanish (*n* = 1) and Italian (*n* = 1); 27 publications that were book chapters (*n* = 5), conference papers (*n* = 5), erratum notices (*n* = 5), letters to the Editor (*n* = 9), publication notes (*n* = 1) and retracted articles (*n* = 2); and 20 publications that dealt with pediatric patients. Hence, out of the 587 outputs, 78 were excluded for the aforementioned reasons, leaving a total of 509 manuscripts included for study in this literature search. It is important to acknowledge that all these manuscripts could not be covered or cited in the context of this narrative review. A flowchart showing our literature search is presented in Figure 1.

## 3. Pathogenesis of SA-AKI

### 3.1. The Roles of Inflammation and Metabolic Reprogramming

**Figure 2:**
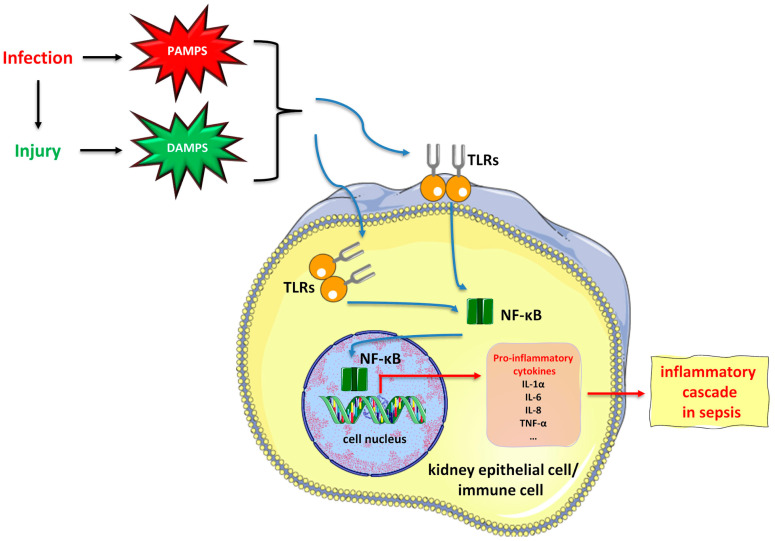
Inflammatory cascade as a component of sepsis in the pathogenesis of SA-AKI. Abbreviations: DAMPs, damage-associated molecular patterns; IL, interleukin; NF-kB, nuclear factor kappa B; PAMPs, pathogen-associated molecular patterns; TLRs, Toll-like receptors; TNF-a, tumor necrosis factor alpha. (All free elements in the figure originated from the free medical site http://smart.servier.com/ by Servier licensed under a Creative Commons Attribution 3.0 Unported License).

Inflammation as an essential component of sepsis seems to play a pivotal role in the pathogenesis of SA-AKI. In particular, PAMPs (pathogen-associated molecular patterns) or DAMPs (damage-associated molecular patterns) may activate TLRs (Toll-like receptors), especially TLR-2 and TLR-4. Apart from being located on the surface of immune cells, TLR-2 together with TLR-4 are also situated on the surface of tubular epithelial cells in the kidney [6,7,11,12]. The activation of TLR-2 and TLR-4 results in an inflammatory cascade, characterized by the release of pro-inflammatory cytokines, such as IL-1α (interleukin-1 alpha), IL-6 (interleukin-6), IL-8 (interleukin-8), and TNF-α (tumor necrosis factor alpha) [12,13]. Furthermore, sepsis alters energy metabolism, resulting in the metabolic reprogramming of immune cells, which consequently deranges innate and adaptive immune responses, causing hyperinflammation and immunosuppression. More specifically, as the energy needs are enhanced in sepsis due to a state of hyperinflammation, a transition from oxidative phosphorylation to glycolysis takes place. Although beneficial at the initial stage of sepsis, metabolic reprogramming results in organ damage at a later stage [6,14,15]. Figure 2 depicts the inflammatory cascade in sepsis.

### 3.2. The Role of Cell Death

#### 3.2.1. Apoptosis

Apoptosis is a type of programmed cell death that is mediated by a caspase cascade resulting in the shrinking of the cell membrane as well as a reduction in the cell’s volume [16,17]. LPS, i.e., lipopolysaccharide of the Gram-negative bacteria, may play a crucial role in SA-AKI-induced apoptosis. Bannerman et al. have suggested that LPS triggers the activation of caspases 3, 6, 7 and 8, through the accumulation of FADD (Fas-associated death domain) and TRADD (TNF-α associated death domain) [18]. The activation of caspase 3 results in the translocation of caspase 3 into the nucleus and, thus, in subsequent DNA degradation, protein hydrolysis and the formation of apoptotic bodies [18]. In addition, the increased production of ROS (reactive oxygen species) further contributes to apoptotic death. More specifically, through the activation of p53 and Bax (B cell lymphoma 2-associated X protein), ROS may lead to mitochondria disruption and their involvement in the apoptotic process [19].

#### 3.2.2. Necroptosis

Necroptosis is a type of regulated cell death that differs significantly from apoptosis. Necroptosis results in an increased cell volume due to the rupture of the cell membrane and the swelling of the organelles. These morphological changes are in sharp contrast to the shrinking of the cell membrane and the cell’s volume seen in apoptosis. Apart from the aforementioned morphological changes, there is no involvement of the caspase cascade in necroptosis [20,21]. On the contrary, other cellular transduction molecules, such as RIPK1 (receptor-interacting protein kinase 1) and RIPK3 (receptor-interacting protein kinase 3), are implicated in necroptosis [20,21]. RIPK1 is activated by binding to TNFR (TNF receptor) and then also activates RIPK3, resulting in the stimulation of MLKL (mixed lineage kinase domain like). The stimulation of MLKL leads to the formation of the oligomers of phosphorylated MLKL. These oligomers are then translocated to the cell membrane, resulting in the destruction of the cell membrane and the necroptotic programmed cell death [20,21].

#### 3.2.3. Pyroptosis

**Figure 3:**
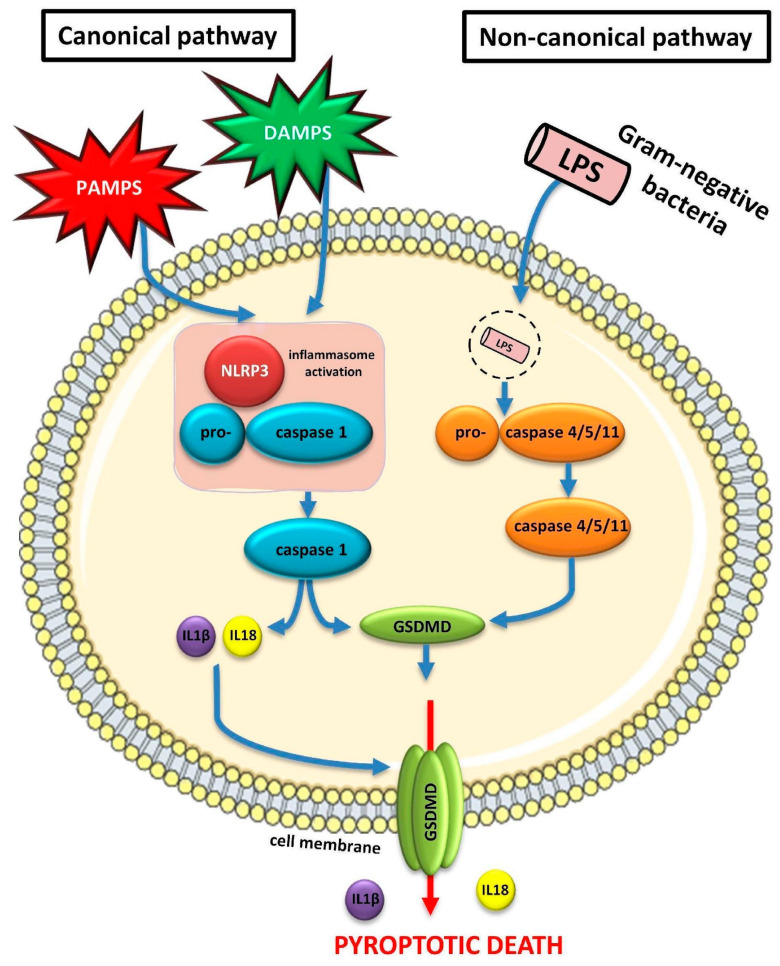
Canonical and non-canonical pathways of pyroptosis implicated in the pathogenesis of SA-AKI. Abbreviations: DAMPs, damage-associated molecular patterns; GSDMD: gasdermin-D; IL, interleukin; LPS: lipopolysaccharide; NLRP3: nucleotide binding domain like receptor protein 3; PAMPs, pathogen-associated molecular patterns. (All free elements in the figure originated from the free medical site http://smart.servier.com/ by Servier licensed under a Creative Commons Attribution 3.0 Unported License).

Pyroptosis is considered a distinct type of regulated cell death that is mediated by the activation of caspase as well as the inflammasome, especially the NLRP3 (nucleotide binding domain like receptor protein 3) inflammasome [22]. The inflammasome is activated by means of two distinct, but interrelated pathways: the canonical and the non-canonical pathway. In the canonical pathway, caspase-1 is implicated. In particular, pro-caspase-1 is activated to form the active caspase-1, which, in turn, activates gasdermin -D (GSDMD). GSDMD possesses the ability to form pores in the cell membrane, thereby causing alterations in the osmotic pressure inside and outside the cell. These changes lead to the swelling of the cell, loss of the integrity of the cell’s membrane and programmed cell death through pyroptosis [22,23]. However, the mitochondria remain intact in the whole process. In the non-canonical pathway, caspase-11 in mice and their human analogs caspase-4 and caspase-5, are activated by the LPS of the Gram-negative bacteria. The activation of caspases-4 and caspases-5 in humans may result in the stimulation of GSDMD, irrespective of caspase-1. Despite the fact that in this pathway only caspase-4 and caspase-5 are being implicated, the result is the same as in the canonical pathway, i.e., the activation of GSDMD and, thus, the formation of pores in the cell’s membrane leading to pyroptotic death [24,25]. Figure 3 depicts the canonical and non-canonical pathways in terms of pyroptotic cell death in SA-AKI.

#### 3.2.4. Ferroptosis

Lately, a newer type of regulated cell death, named ferroptosis has been described. It is related to an excess iron aggregation in the cell, which may lead to an increased production as well as a decreased elimination of lipid peroxides [26,27,28]. In this context, a dysregulation in the metabolism of cellular glutathione may result in an iron-dependent programmed cell death known as ferroptosis [26,27,28]. Despite the fact that ferroptosis has been associated with brain and heart diseases, its relationship to kidney disorders has not been established definitively. However, until today, there are a few reports associating ferroptosis with SA-AKI [17,26]. In addition, glutathione peroxidase 4 (GPX4) as well as prostaglandin endoperoxide synthase 2 (PTGS2) have been proposed as biomarkers of ferroptosis. Notably, Xiao et al. demonstrated decreased levels of GPX4 in conjunction with increased concentrations of PTGS2 in SA-AKI [26]. Nevertheless, the pathogenetic mechanisms of ferroptosis are not yet fully elucidated, especially in SA-AKI.

### 3.3. Autophagy and Efferocytosis

Autophagy or autophagocytosis is a complex process that is used by the cell to remove any unnecessary components and, thus, to promote cell homeostasis. Autophagy is accomplished through the formation of autophagosomes and through the subsequent fusion of autophagosomes with lysosomes to form a mature phagolysosome [29,30,31]. Within the acidic background of the mature phagolysosome, any unnecessary components, such as microbes or degrading organelles, are destroyed and removed. In this complicated procedure, which is lysosome-dependent, a significant number of signaling molecules are implicated. Although the exact mechanisms are not yet completely understood, there seems to be an interplay between the mTOR (mammalian target of the rapamycin), sirtuins, especially sirtuins 1, 3 and 6, and NF-*κ*B (nuclear factor kappa B) [31,32,33]. As a result of cellular stress, autophagy is being regulated by autophagy-related genes (ATG), such as *Atg5* and *Atg7*, which have already been documented to be involved in the development of kidney disease [34,35]. In addition, Zhao et al. have already proposed the complex interplay between ATG and the mTOR, sirtuins 1, 3 and 6, and the NF-*κ*B signaling pathways in SA-AKI [32]. Autophagy may play a crucial role in the pathogenesis of SA-AKI [29,30,31,32,33,34,35]. Most studies are in agreement with the notion that autophagy rises early in the development of SA-AKI and falls afterward [29,30,31,32,33,34,35]. Moreover, autophagy has been proposed to be a key player in SA-AKI therapeutics and could serve as a major target for SA-AKI prevention and treatment [29,30,31,32,33,34,35]. Apart from autophagy, another process called “efferocytosis” could also be involved in SA-AKI. Efferocytosis is defined as the “clearance of dying or dead cells from professional and non-professional phagocytes” [36]. In order to avoid the excess formation of DAMPs, the removal of dead cells should be a fast procedure. Otherwise, DAMPs may accumulate, resulting in hazardous effects. In this context, the apoptosis inhibitor of macrophages (AIM) has been shown to play a pivotal role. More specifically, the AIM binds to IgM, leading to the inhibition of its renal excretion. On the contrary, during AKI, the AIM is separated from IgM, being filtered in the glomeruli. In the kidney, the AIM binds to kidney injury molecule-1 (KIM-1), thus promoting SA-AKI through efferocytosis [36,37,38]. The AIM may represent a potential novel therapeutic target in SA-AKI. 

### 3.4. The Role of Hemodynamic Changes

Macrovascular as well as microvascular dysfunction in SA-AKI are likely to be equally involved in the pathogenesis of SA-AKI. Macrovascular changes mainly caused by hypotension in septic shock and a decreased glomerular filtration rate remain, thus far, the cornerstone of treatment in SA-AKI. Generally, the use of vasoconstrictors to maintain a MAP over 65 mmHg has been associated with an improvement in organ dysfunction. Many studies have shown that the restoration of MAP may improve the urinary output and renal indices as well [39,40,41,42]. Therefore, the amelioration of MAP remains a common clinical practice in the prevention and treatment of SA-AKI [39,40,41,42]. However, apart from the use of intravenous fluids and vasopressors to maintain a normal renal blood flow, the hosts’ physiological rebound mechanisms, such as the RAAS (renin angiotensin aldosterone system) and the activation of the sympathetic nervous system are also implicated. It is noteworthy that except for macrocirculation alterations in SA-AKI, the involvement of microcirculation dysfunction is suggested to be equally significant in the development of SA-AKI [41,42]. In particular, the roles of pro-inflammatory cytokines, the extracellular matrix and cell adhesion molecules are being increasingly recognized, nowadays. The aggregation of the aforementioned factors in conjunction with the activation of platelets may result in the formation of microthrombi in the microvascular circulation. The increased production of ROS and iNOS (inducible nitric oxide synthase), the latter catalyzing the release of NO (nitric oxide), have been implicated in microcirculatory dysfunction [40]. In addition, the mitigation of blood flow in the capillaries is also accompanied by capillary leakage and subsequent interstitial edema in the kidneys, which may appear even within the first 2 h of sepsis [42,43]. Overall, macrovascular as well as microcirculatory parameters seem to play an important role in the pathogenesis of SA-AKI.

## 4. Biomarkers in SA-AKI

Table 1 depicts major biomarkers in SA-AKI. Apart from urine output and serum creatinine levels, which have been incorporated in the definition of AKI, other biomarkers have also been developed. In serum, on account of their shorter half-lives than serum creatinine, cystatin C levels and plasma proenkephalin A 119–159 (penKid) are increasingly being used [44]. Serum cystatin C has already been used in CKD-EPI (chronic kidney disease epidemiology collaboration) equations, whereas penKid is a member of the enkephalins group, with concentrations in CSF (cerebrospinal fluid) 100 times higher than its serum concentrations. Serum penKid is lately considered an earlier marker of AKI than serum creatinine. In addition, although its serum concentrations seem to be unaffected by inflammatory mechanisms, serum penKid has been advocated as a biomarker of early SA-AKI [45,46]. Other potential serum biomarkers of SA-AKI are interleukins, such as IL-6 (interleukin-6) and IL-8 (interleukin-8), the cytokine osteoprotegerin, galectin-3, and presepsin [5,47,48,49,50]. IL-6 and IL-8 are elevated in patients with SA-AKI, while urinary levels of IL-18 may be representative of kidney damage and have recently been shown to be elevated in SA-AKI [51]. Osteoprotegerin is a cytokine receptor of the TNF superfamily, which has been documented to bind to TNF-related apoptosis-induced ligand (TRAIL), thereby inhibiting its action. Osteoprotegerin may be a reliable marker of SA-AKI, with a significant correlation to cystatin C and KIM-1. However, more studies are needed to confirm its diagnostic and prognostic value as a biomarker of SA-AKI [47]. Galectin-3 is a glycan-binding protein with pleiotropic properties that has been demonstrated to be involved in the pathogenesis of SA-AKI. In particular, galectin-3 can be recognized by TLR-4, while it has the ability to activate the caspase 4/11 pyroptotic pathway to augment inflammation in sepsis. Its inhibition seems to be very promising, as it may prevent the non-canonical inflammasome pathway, which may lead to kidney damage in sepsis [50]. Furthermore, presepsin is a glycoprotein expressed on the surface of monocytes and macrophages. Its serum levels increase as a result of activation by microorganisms. Therefore, it is considered a relatively novel, albeit promising biomarker of sepsis and SA-AKI. Presepsin levels are increased in SA-AKI [48].

In urine, dipstick albuminuria together with urine microscopy have been a useful tool for a long time, while the urine levels of KIM-1, NGAL (neutrophil gelatinase-associated lipocalin), CCL-14 (chemokine ligand 14), TIMP-2 (tissue inhibitor of metalloproteinase 2), PTGS2 (Prostaglandin endoperoxide Synthase 2) and sTREM-1 (soluble triggering receptor expressed on myeloid cells 1) are being tested as potential urinary biomarkers of SA-AKI [5,52,53,54]. Some experts have proposed the use of urinary L-type fatty acid binding protein (L-FABP) as a biomarker of SA-AKI. Urinary concentrations of L-FABP have been associated with renal damage in the context of various disorders, including sepsis. Even though serum levels of L-FABP were initially found to be related to liver damage, its increased urinary levels have been associated with a degree of renal damage during sepsis [5,55]. 

Apart from serum and urinary biomarkers, there is ongoing research regarding molecular biomarkers of SA-AKI. In particular, gene expression profiles in SA-AKI together with biomarkers stemming from proteomics and metabolomics have revolutionized the field of SA-AKI in terms of diagnosis and prognosis [56,57,58,59,60,61,62]. Regarding gene expression profiles, Guo et al. have very recently demonstrated that the *AFM* gene, also called “afamin”, which is a member of the albumin family of genes, may be a surrogate biomarker for SA-AKI. More specifically, using the Gene Expression Omnibus (GEO) Synthesis database and Weighted Gene Co-expression Network Analysis (WGCNA), they documented afamin as a biomarker of SA-AKI being inversely associated with the recruitment of monocytes and the levels of various inflammatory factors [57]. By using the GEO database as well, Liu et al. found that 7 out of the 15 hub genes are involved in the enriched Kyoto Encyclopedia of Genes and Genomes (KEGG) pathway. The 7 implicated genes are *Hmox1*, *Spp1*, *Socs3*, *Mapk14*, *Lcn2*, *Cxcl1* and *Cxcl12*. In addition, the mmu-miR-7212-5p-Hmox1 RNA pathway was shown to play a crucial role in the RNA regulatory axis in ferroptosis. *Hmox1* denotes heme oxygenase 1, and has been implicated in ferroptosis, which, in turn, is activated in SA-AKI, as already mentioned. Furthermore, N-6 adenosine methylation, m6A and RNA methylation modifications were shown to be involved in SA-AKI [58]. Moreover, Ma et al. proposed that the reduction in the expressions of miR-370-3p and miR-495-3p in the urine of patients with SA-AKI are biomarkers in SA-AKI [59]. Very recently, apart from non-coding RNAs, Ma et al. have studied the role of circular RNAs in the development of SA-AKI. They have reported that the circFkbp5/miR-760-3p/TNF-α axis is fundamental in SA-AKI and may serve as a potential therapeutic target as well [60]. With the advent of bioinformatics, machine learning models have been introduced to predict prognosis in SA-AKI. For example, Luo et al. employed a computerized program named eXtreme Gradient Boosting (XGBoost) that may perform better than the SOFA (sequential organ failure assessment) score for the timely identification of patients with a worse prognosis of SA-AKI [63]. They used this program on 15,873 patients with sepsis, among whom 12,132 patients had SA-AKI [63]. The same machine learning algorithm outperformed the SOFA score in the study by Li et al. among 8129 patients with sepsis regarding mortality prediction from SA-AKI [64]. Previously, Yang et al. had suggested the use of a nomogram that they had tested among 2871 patients with sepsis, which they had developed using a multivariate regression analysis model [65]. Overall, it seems likely that mathematical and machine program algorithms are very useful tools in the early diagnosis and better management of patients with SA-AKI. Interestingly, these algorithms may help to timely treat patients with worse prognoses.

**Table 1:** Major biomarkers associated with SA-AKI.

Biomarkers	Material	Features	References
**Circulating**			
Creatinine	Serum/Plasma	Byproduct of creatine phosphate catabolism, inverse correlation with GFR Most used biomarker of kidney function Creatinine is used in the definition and staging of AKI	[5]
Cystatin C	Serum/Plasma	Inverse correlation with GFR, shorter plasma half-life than creatinine; their combination likely reflects GFR more accurately	[5]
Proenkephalin A (penKiD)	Serum/Plasma	Stable peptide of the enkephalin family, inverse correlation with GFR	[44,45,46]
Interleukin-6	Serum/Plasma urine	Secreted by immune cells (macrophages/monocytes) and possibly renal resident cells under certain conditions, elevated early in SA-AKI	[5]
Interleukin-8	Serum/Plasma	Potent neutrophil chemoattractant, produced by, among others, glomerular, tubular and interstitial renal cells, elevated early in SA-AKI	[5]
Osteoprotegerin	Serum/Plasma	Elevated in the CKD and AKI of various causes, including SA-AKI	[47]
Galectin-3	Serum/Plasma	Elevated in AKI, including early increases in SA-AKI	[49,50]
Presepsin	Serum/Plasma	Increases in serum/plasma are predictive of AKI in patients with sepsis Increase in serum during the early stages of sepsis (≈2 h after bacterial infections) Presepsin differentiates sepsis from surgical operations, trauma and burn injuries	[48]
**Urinary**			
Urine output (UO)	Urine	A cutoff of UO < 0.5 mL/kg/h for 6 h is implemented in the Kidney Disease: Improving Global Outcomes (KDIGO) diagnostic criteria for AKI; the trajectory of UO within 24 h of sepsis onset is associated with the risk of SA-AKI	[4]
Dipstick albumin	Urine	Wide and practical screening tool for the assessment of kidney disease	[5]
Microscopy	Urine	Wide, practical but not validated test used to differentiate the etiology of kidney disease A non-validated score based on the number of renal tubular epithelial cells and/or the granular casts per high-powered field has been suggested for SA-AKI Urine microscopy is subjected to inter-observer variability	[4,5]
Kidney Injury Molecule-1 (KIM-1)	Urine	Type 1 transmembrane protein, increased levels in urine in proximal tubular injuries, including SA-AKI	[51,52,53,54]
Neutrophil gelatinase-associated lipocalin (NGAL)	Urine	Released from neutrophil granules as well as renal tubular stress after injury, increased urine concentrations in SA-AKI	[51,52,53,54]
Chemokine (C-C motif) ligand 14 (CCL-14)	Urine	Chemokine produced by renal tubular cells in AKI, urinary levels are predictive of persistent SA-AKI	[51,52,53,54]
[TIMP-2] × [IGFBP7]	Urine	The product of cell-cycle arrest biomarkers Tissue inhibitor of metalloproteinases-2 and Insulin-like growth factor binding protein 7 ([TIMP-2] × [IGFBP7]) predicts SA-AKI and 30-day mortality in affected patients	[5,51,52,53,54]
Prostaglandin endoperoxide Synthase 2/Cyclooxygenase-2 (PTGS2)	Urine	Biomarker of ferroptosis, increased in urine during SA-AKI	[51,52,53,54]
Soluble triggering receptor expressed by myeloid cells 1 (sTREM-1)	Urine	Elevated in urine early in the course of SA-AKI, potentially as a consequence of the increased local production by renal resident cells	[51,52,53,54]
L-type fatty acid binding protein (L-FABP)	Urine	Increases in urine precede serum creatinine elevations during SA-AKI	[5,55]
miR-370-3p miR-495-3p	Urine	In patients with sepsis, the decreased expressions of microRNAs miR-370-3p and miR-495-3p distinguish those with SA-AKI and are associated with poorer prognoses	[59]
Circular RNA particles	Urine	Several circular RNAs undergo up- (e.g., hsa_circ_0040994/circ-FANCA) or down-regulation (e.g., hsa_circ_0068,888) in SA-AKI	[60]
**Molecular**			
AFM gene expression	Kidney tissue, monocytes	Decreased expression and negative correlation with monocyte infiltration	[57]
Differentially expressed genes in kidney tissue	Kidney tissue	Differential expression of several hub genes (Hmox1, Spp1, Socs3, Mapk14, Lcn2, Cxcl1 and Cxcl12) in a mouse model of SA-AKI	[57,58]
Post-transcriptional mRNA modifications	Kidney tissue	m6A RNA methylation involved in SA-AKI in a mouse model	[58]

## 5. Prevention of SA-AKI

The prevention of SA-AKI may be difficult in the clinical setting, as many patients presenting in the emergency department may already have septic shock or SA-AKI. Nevertheless, the timely administration of antibiotics together with source control of the infection seem to be the most significant measures in preventing the development of SA-AKI. Moreover, the initiation of intravenous fluids and the maintenance of an adequate MAP are very important as well. In this context, the use of vasopressors may also be helpful. Notably, as the current definition of SA-AKI was introduced only recently (in 2023), more studies, including randomized controlled studies and meta-analyses, are anticipated to further improve our knowledge on the prevention of SA-AKI [5,66].

## 6. Therapeutics of SA-AKI: Current Knowledge and Future Perspectives

In 2023, the 28th ADQI Group reported that a balanced fluid resuscitation to further redistribute the intracellular volume together with the as-soon-as-possible administration of appropriate antibiotics to combat sepsis are of the utmost importance [5]. However, the role of measuring central venous pressure (CVP) in this context does not seem to be clear. CVP measurement is a rather rough estimation of fluid overweight in the body, and it is influenced by the right ventricular function. Therefore, the fluid challenge and assessment of fluid responsiveness seem to be more helpful than CVP measurements in the management of fluid administration to avoid fluid depletion or fluid overload [5,67,68,69]. Nevertheless, the choice of which fluids should be administered, for example, 0.9% saline versus colloids, depends upon various parameters. In early SA-AKI, a more aggressive fluid administration to restore hemodynamic stabilization seems mandatory, whereas in late SA-AKI, the main concern for clinicians is fluid overload [5,67,68,69,70,71,72,73]. Apart from this major difference between the needs in early versus late SA-AKI, a more personalized approach, bearing in mind the performance status of each patient as well as comorbidities, should be pursued. The use of diuretics may be individualized as well. Regarding the administration of more specified fluids, such as hydroxyethyl starch, a synthetic colloid made of amylopectin, there is consensus that it should be avoided, as it has been related to an increased mortality and increased KRT in patients with sepsis [5,67,68,69,70,71,72,73]. However, in terms of supplementation with albumin, the results are controversial. For the time being, the results of the Albumin Replacement Therapy In Septic Shock (ARISS) Trial are much awaited to further shed light upon this issue [70]. This trial’s methodology was published in 2020, but its results have not been published yet.

Regarding KRT, the timing of the initiation of KRT, i.e., early versus late, is still a matter of debate. Moreover, the “early” or “late” timings are not strictly defined. Therefore, although there is a significant number of studies advocating for the “early” initiation of KRT, there are also many studies favoring the “late” initiation of KRT [74,75,76]. Despite the fact that the indications for KRT are well known, such as severe hyperkalemia and acidosis, anuria, fluid overload and uremic pericarditis or encephalopathy, the aforementioned estimations are sometimes individualized and should be personalized as well. Although the “early” versus “late” timing of KRT initiation seems to play a pivotal role, this may not be really true and may just reflect the clinicians’ anxiety or the lack of a unifying language among them. In addition, more significant seems to be the choice of which extracorporeal blood purification (EBP) method to use in cases of SA-AKI. It is widely known that different EBP methods result in the elimination of different pathogens, endotoxins and inflammatory parameters. For example, polymyxin B hemoperfusion has lately been used with inconclusive results. An ongoing trial, the TIGRIS trial is under investigation for its positive or negative outcomes regarding patients with SA-AKI [77,78]. Moreover, synthetic resins may allow for the removal of excessive pro-inflammatory factors leading to the avoidance of the cytokine storm observed in sepsis. Therefore, the choice and perhaps the timing of the EBP method to follow should be performed by well-trained personnel in patients with SA-AKI [5,79,80,81,82,83].

Apart from these general measures, the involvement of the RAAS in the pathogenesis of SA-AKI, with dysregulation especially of the angiotensin II/angiotensin II receptor 1 axis (AT-1R), has been documented [84,85,86]. Garcia et al. have suggested that among patients with high renin levels and sepsis, the administration of angiotensin II could reduce renin levels and ameliorate the intra-renal hemodynamics. Thus, they have proposed that the administration of angiotensin II among patients with high renin levels and SA-AKI, may present beneficial effects to this category of patients [84,85,86]. Except for the RAAS, the development of other potential biomarkers, based upon the pathophysiology of SA-AKI is under consideration. For example, regarding autophagy, the presence of long non-coding RNAs (lncRNAs) as well as microRNAs (miRNAs), such as PlncRNA-1, miR-22-3p, miR-526b, LncRNA NKILA, miR-140-5p and miR-214, has been documented to be involved in SA-AKI. Therefore, according to Zhao et al., the abovementioned lncRNAs and miRNAs could serve as therapeutic targets [32]. In addition, Gao et al. have proposed that the compound polydatin, through the activation of SIRT-1 and the inhibition of NLRP3 activation, could protect against mitochondrial dysfunction [87]. More specifically, they have documented that polydatin could exert beneficial effects on mitochondrial dysfunction by activating Parkin-dependent autophagy [87]. In addition, Chen et al. have found that ascorbate could improve LPS-induced SA-AKI by interfering with the PINK-1/PARK-2 pathway [88]. Furthermore, Jia et al. have suggested that alpha lipoic acid through the up-regulation of autophagy genes, such as *Atg5*, *Atg 7* and *Beclin-1*, may ameliorate renal function in an animal model of SA-AKI [89]. Very recently, Guo et al. identified seven ferroptosis genes implicated in SA-AKI. More specifically, *OLFM4*, *CLU*, *RRM2* and *SLC2A3* were up-regulated in an LPS-induced animal SA-AKI model, whereas *CCL5*, *ADAMTS1* and *EPHX2* were down-regulated in this animal model [90]. They also advocated for the use of Tregs as well as salubrinal for the treatment of SA-AKI [91]. Tregs are T cells that possess anti-inflammatory and immuno-protective properties, while salubrinal is a compound that inhibits the dephosphorylation of eukaryotic translation initiation factor 2α, (eIF2-α), thus protecting the cell from ER stress [90,91]. Table 2 shows major studies regarding the potential uses of various therapeutic targets as a means to combat SA-AKI.

Notably, there is growing interest in the classification of sub-phenotypes among patients with SA-AKI [5,71]. Recently, Lai et al. proposed the existence of three different sub-phenotypes in patients with SA-AKI [71]. They have suggested that sub-phenotype 1 involved younger patients, with higher baseline levels of the estimated glomerular filtration rate (eGFR) and a lower Charlson Comorbidity Index (CCI) [71,92]. In their study, Lai et al. used the CCI Calculator, which comprises a series of comorbidities, such as cardiovascular disease, renal disease, diabetes, etc. and provides a relevant score [71,92]. However, sub-phenotype-1 patients with SA-AKI had a higher risk of mortality and a higher risk of chronic dependence on KRT when compared to sub-phenotype 3 patients with SA-AKI [71]. Moreover, a serum level of lactate > 3.3 mmol/L before the initiation of KRT was related to worse outcomes. This level of hyperlactatemia was associated with an increased mortality and a higher risk of chronic dependence upon KRT [71]. Nevertheless, the categorization of patients with SA-AKI into sub-phenotypes seems to be a demanding procedure and has a long way ahead [93].

**Table 2:** List of main studies on animal models associating potential therapeutic targets with SA-AKI pathogenetic mechanisms.

Research/Year	Population, Type of Study	Treatment	Main Findings	Remarks
Howell et al., 2013 [94]	C57Bl/6 mice 8 ws (young) and 45 ws (adult) old were administered LPS 1.5 mg/Kg ip. to induce endotoxemia	Temsirolimus 5 mg/Kg iv. (Inhibitor of mTOR)	Autophagy was documented in this SA-AKI animal model.	✓ The administration of temsirolimus, an inhibitor of mTOR, resulted in an amelioration in renal function even in adult mice.
Sunahara et al., 2018 [95]	C57Bl/6 male mice 6–8 ws old and microtubule-associated protein light chain 3 (LC3) transgenic mice (C57BL/6 background) 6–8 ws old underwent CLP procedure	Rapamycin 10 mg/Kg ip. (Inhibitor of mTOR)	Autophagy was related to an improvement in the histological findings in terms of tubular epithelial injury in mice.	✓ Rapamycin induced autophagy and resulted in an improvement in the renal histopathological findings, but not in serum creatinine levels.
Jia et al., 2019 [89]	Male Sprague–Dawley rats underwent CLP procedure	ALA 200 mg/Kg via oral gavage	ALA increased the expressions of *Atg5*, *Atg7* and *Beclin1*, i.e., autophagy-related genes.	✓ ALA enhanced autophagy through the formation of autophagosomes in the kidneys of SA-AKI rats.
Gao et al., 2020 [87]	Adult male and female C57BL/6 mice underwent CLP procedure	PD 30 mg/Kg iv	PD induced the translocation of Parkin from the cytoplasm to the mitochondria in SA-AKI mice.	✓ PD increased Parkin-induced mitophagy through the activation of SIRT1 as well as the inactivation of NLRP3.
Liu et al., 2020 [96]	Mice were administered LPS 10 mg/Kg ip. to induce SA-AKI	Procyanidin B2	Procyanidin B2 improved autophagy by increasing the nuclear translocation of the transcription factor NRF2.	✓ Procyanidin B2 improved mitophagy, i.e., mitochondrial autophagy in SA-AKI mice treated with Procyanidin B2.
Miao et al., 2020 [97]	Male C57BL/6 8–10 ws old were administered LPS 10 mg/Kg ip.	SW033291, 10 mg/Kg ip. (Small-molecule inhibitor of 15-PGDH)	The inhibition of 15-PGDH resulted in reduced expressions of Fas, caspase-3 and caspase-8.	✓ SW033291 administration improved SA-AKI in mice by ameliorating autophagy, apoptosis and oxidative stress.
Yang et al., 2020 [98]	Wistar rats were administered LPS 10 mg/Kg ip.	DEX 30 μg/Kg ip.	DEX had nephro-protective effects by means of the AMPK/mTOR pathway. The administration of atipamezole and methyladenine reversed the beneficial effects of DEX.	✓ DEX improved autophagy and inhibited the activation of NLRP3.
Feng et al., 2020 [99]	Male Sprague–Dawley rats 8 ws old were administered LPS to induce SA-AKI	HUCBMNC in a final concentration of 3 × 10^7^ MNCs/mL that mainly expressed CD3, CD38 and CD5 positivity	The activation of the NRF2 pathway decreased inflammation and ameliorated autophagy.	✓ HUCBMNCs seem to improve SA-AKI by means of increasing the expression of the NRF2 pathway.
Zhao et al., 2020 [100]	Male adult Sprague–Dawley rats were administered 10 mg/Kg LPS ip.	DEX 30 μg/Kg ip.	DEX improved SA-AKI. The administration of atipamezole and MA reversed the beneficial effects of DEX.	✓ DEX resulted in the inhibition of the PI3K/AKT/mTOR axis and, thus, in decreases in mitochondrial damage and oxidative stress.
Guo et al., 2021 [101]	Female Sprague–Dawley rats 12 ws old underwent CLP procedure	BMSCs (5 × 10^6^ cells/mL)	BMSCs resulted in improvement in SA-AKI.	✓ Improvement in SA-AKI was accomplished through the activation of the SIRT1/Parkin pathway, which led to decreased pyroptosis and increased mitophagy.
Tan et al., 2021 [102]	60 BALB/c male mice 6–8 ws old underwent CLP procedure	2-DG 2 g/Kg ip (Inhibitor of aerobic glycolysis)	2-DG resulted in the amelioration of SA-AKI.	✓ 2-DG, by inhibiting aerobic glycolysis, enhances autophagy by means of the activation of the SIRT3/AMPK axis.
Li et al., 2022 [103]	140 male C57BL/6 mice 8 ws old were administered LPS 10 mg/Kg ip.	H_2_S (NaHS) 0.8 mg/Kg ip.	Exogenous H_2_S improved SA-AKI.	✓ Improvement in SA-AKI was achieved through the activation of autophagy. The beneficial effects of H2S were decreased through the administration of 3-MA, an inhibitor of autophagy.
Li et al., 2022 [104]	Sprague–Dawley male adult rats	Ulinastatin 5000 U/Kg iv (Urinary trypsin inhibitor)	Unilastatin improved SA-AKI.	✓ Improvement was attributed to the decreased production of cytokines and the amelioration of microcirculation in the kidneys of SA-AKI rats.
Yu et al., 2022 [105]	Mice model of SA-AKI were adminstered 10 mg/Kg of LPS ip.	Oral administration of NF-*κ*B inhibitor 270	Inhibitor 270 resulted in an improvement of SA-AKI.	✓ Improvement was achieved through the inhibition of the NF-*κ*B and JNK axis, thus reducing inflammatory factors.
Luo et al., 2023 [106]	Male C57BL/6 mice 6–8 ws old underwent CLP procedure	MSCs 1 × 10^6^ cells iv + Gal-9 (100 μg/mouse) iv)+ Soluble Tim-3 (100 μg/mouse) iv	MSCs altered the Th1/Th2 ratio and achieved cellular homeostasis. However, when soluble Tim-3 was administered, it resulted in blocking the Gal-9/Tim-3 axis and, thus, in increased mortality from SA-AKI.	✓ MSCs improved immune-cells’ homeostasis through the activation of the Gal-9/Tim-3 axis.
Zhang et al., 2023 [107]	Male C57BL/6 mice underwent CLP procedure	Exogenous H_2_S (GYY4137) administered to SA-AKI mice	Exogenous H_2_S improved SA-AKI by inhibiting ferroptosis.	✓ H2S reduced ferroptosis markers and mitochondrial oxidative stress.
Luo et al., 2023 [108]	C57BL/8 mice underwent CLP procedure	MSCs × 10^6^ cells iv + Gal-9 (100μg/mouse) iv)+ Soluble Tim-3 (100μg/mouse)	MSCs inhibited Th17, wich promoted the expansion of Tregs.	✓ MSCs improved SA-AKI by promoting Tregs and inhibiting Th17 differentiation.
Yang et al., 2024 [109]	Mice model of SA-AKI were injected with LPS ip.	PCE was administered to SA-AKI mice	PCE resulted in a reduction in the production of inflammatory cytokines.	✓ Emodin and PD, as ingredients of PCE, mitigated the action of inflammatory cytokines through the inactivation of NF-*κ*B.

Abbreviations: ALA: Alpha Lipoic Acid; AMPK: 5- Adenosine Monophosphate-activated Protein Kinase; BMSCs: Bone Marrow derived Mesenchymal Stem Cells; CLP: Cecal Ligation and Puncture; 2-DG: 2 Deoxy-Glucose; DEX: dexmedetomidine; hUCBMNCs: human Umbilical Cord Blood Mononuclear Cells; IP: Intraperitoneally; IV: Intravenously; JNK: c-Jun N-terminal Kinase; LPS: Lipopolysaccharide; 3-MA: 2-Methyladenine; MNCs: Mononuclear Cells; MSCs: Mesenchymal Stem Cells; mTOR: mammalian Target of Rapamycin; NF-*κ*B: Nuclear Factor kappa B; NLRP3: Nucleotide binding domain-like receptor protein 3 inflammasome; NRF2: Nuclear Factor Erythroid 2 Related Factor 2; PCE: Polygonum Cuspidatum Extract; PD: Polydatin; 15-PGDH: 15-Hydroxyprostaglandin Dehydrogenase; Tregs: T regulatory cells; ws: weeks.

## 7. Conclusions

SA-AKI remains a difficult clinical entity that clinicians must confront, with a substantial morbidity and mortality, above and beyond the existence of solely sepsis or AKI. Its pathogenesis includes the complex interplay between several inflammatory factors, macrovascular as well as microvascular dysfunction, apoptosis, necroptosis, pyroptosis, ferroptosis, autophagy and efferocytosis. The pathogenetic mechanisms underlying SA-AKI, especially in terms of regulated cell death forms, are being studied elaborately. However, further research is needed to elucidate the exact pathways involved. In this context, multi-omics techniques are expected to shed light upon the intricate interconnections between various pathogenetic pathways. The presence of long non-coding RNAs (lncRNAs) as well as microRNAs (miRNAs), such as PlncRNA-1, miR-22-3p, miR−526b, LncRNA NKILA, miR-140-5p and miR-214, which are demonstrated to be involved in the pathogenesis of SA-AKI, may serve as drug targets in the future. The combination of omics technologies represents an innovative holistic approach toward providing a more integrated view of the molecular and physiological events underlying SA-AKI as well as for deciphering unique and specific phenotypes. Although more evidence is still necessary, it is expected that the incorporation of integrative omics may be useful not only for the early diagnosis and risk prognosis of SA-AKI, but also for the development of potential therapeutic targets that could revolutionize the management of SA-AKI for a multidisciplinary team. Therefore, in the future, a more personalized approach depending on the findings from multi-omics studies and bioinformatics should be pursued in the monitoring and therapeutics of SA-AKI and its complications. 

## Data Availability

Not applicable.

## Abbreviations

ADQI: Acute Disease and Quality Initiative; AFM: Afamin; AKI: Acute Kidney Injury; AMPK: 5-adenosine Monophosphate Protein Kinase; ARISS: Albumin Replacement Therapy In Septic Schock; AT-1R: Angiotensin II Receptor 1; ATG: Autophagy related Genes; Bax: B cell lymphoma 2-associated X protein; CCI: Charlson Comorbidity Index; CKD-EPI: Chronic Kidney Disease Epidemiology Collaboration; CCL14: Chemokine Ligand 14; CSF: Cerebrospinal Fluid; CVP: Central Venous Pressure; DAMPs: Damage Associated Molecular Patterns; EBP: Extracorporeal Blood Purification; eGFR: estimated Glomerular Filtration Rate; ER: Endoplasmic Reticulum, FADD: Fas-Associated Death Domain; GEO: Gene Expression Omnibus; GSDMD: Gasdermin-D; GPX4: Glutathione Peroxidase 4; IL: Interleukin; iNOS: inducible Nitric Oxide Synthase; KDIGO: Kidney Disease Improving Global Outcomes; KEGG: Kyoto Encyclopaedia of Genes and Genomes; KIM-1: Kidney Injury Molecule 1; KRT: Kidney Replacement Therapy; lncRNAs: long non-coding RNAs; LPS: Lipopolysaccharide; MAP: Mean Arterial Pressure; miRNAs: microRNAs; MLKL: Mixed Lineage Kinase domain Like; mTOR: mamalian Target Of Rapamycin; NF-*κ*BP: Nuclear Factor kappa B; NGAL: Neutrophil Gelatinase Associated Lipocalin; NLRP3: Nucleotide binding domain-Like Receptor Protein 3 inflammasome; NO: Nitric Oxide; PAMPs: Pattern Associated Molecular Patterns; PTGS2: Prostaglandin endoperoxide Synthase 2; RAAS: Renin Angiotensisn Aldosterone System; RIPK1: Receptor Interacting Protein Kinase 1; RIPK3: Receptor Interacting Protein Kinase 3; ROS: Reactive Oxygen Species; SA-AKI: Sepsis associated Acute Kidney Injury; SIRT: Sirtuins; SOFA: Sequential Organ Failure Assessment; sTREM-1: soluble Triggering Receptor Expressed on Myeloid cells 1; TIMP-2: Tissue Inhibitor of Metalloproteinase 2; TLR: Toll-Like Receptors; TNF-a: Tumor Necrosis Factor alpha; TRADD: TNF-α Associated Death Domain; TNFR: Tumor Necrosis Factor Receptor; WGCNA: Weighted Gene Co-expression Network Analysis; XGBoost: eXtreme Gradient Boosting.
